# The Translational Landscape Revealed the Sequential Treatment Containing ATRA plus PI3K/AKT Inhibitors as an Efficient Strategy for AML Therapy

**DOI:** 10.3390/pharmaceutics14112329

**Published:** 2022-10-28

**Authors:** Ke Wang, Ziyao Ou, Ge Deng, Shufang Li, Jingjing Su, Yayun Xu, Renpeng Zhou, Wei Hu, Feihu Chen

**Affiliations:** 1 School of Pharmacy, Anhui Medical University, Hefei 230032, China; 2 Inflammation and Immune Mediated Diseases Laboratory of Anhui Province, Anhui Institute of Innovative Drugs, Hefei 230000, China; 3 Anhui Province Key Laboratory of Major Autoimmune Diseases, Anhui Medical University, Hefei 230032, China; 4 Department of Clinical Pharmacology, The Second Hospital of Anhui Medical University, Hefei 230601, China

**Keywords:** ATRA, AML, translatome, Ribo-seq, PI3K/AKT, eIF4E

## Abstract

The present study aimed to better understand the possibility of utilizing all-trans retinoic acids (ATRA) in acute myeloid leukemia (AML). We found that ATRA significantly suppressed global translation and protein synthesis in AML cells. The efficacy of ATRA in treating AML required its translational regulatory functions, as shown by the fact that the decrease in the universal eukaryotic initiation factor 4E (eIF4E) was essential to maintain the induction of cell growth arrest and differentiation by ATRA. By establishing a specific translational landscape, we suggested that transcripts with simple 5′UTR gained a translational advantage in AML cells during ATRA stress. Based on that, the genes translationally regulated by ATRA were mainly enriched in phosphatidylinositol-3-kinase/Akt (PI3K/AKT) signaling; we subsequently revealed that PI3K/AKT activation was required for ATRA to effectively induce AML cell differentiation. However, PI3K/AKT has been reported to promote the stemness of AML cells. As such, we further suggested that sequential treatment including ATRA and PI3K/AKT inhibitor induced robust apoptosis, extremely inhibited the clonality of AML cells, and suppressed the FMS-like tyrosine kinase 3-internal tandem duplication (FLT3-ITD)-driven transformation of CD34^+^ hematopoietic stem/progenitor cells. Future clinical studies are warranted to further support the clinical application of the sequential strategy for the effective treatment of AML.

## 1. Introduction

Acute myeloid leukemia (AML) is a life-threatening hematopoietic malignancy originating from transformed leukemia stem cells (LSCs), characterized by uncontrolled proliferation, rapid self-renewal, and impaired differentiation [1,2]. Representative standard AML therapies include intensive combination chemotherapy and hematopoietic cell transplantation, both of which are confronted with challenges. The majority of AML patients still ultimately succumb to the disease, their relapse due to residual drug-resistant LSCs and their progeny [3]. All-trans retinoic acid (ATRA), as a single chemotherapy or in combination with other agents, is a very promising way towards successful therapy for AML [4]. However, except for acute promyelocytic leukemia (APL), a subtype of AML, ATRA failed to achieve complete remission (CR) in patients with other subtypes of AML [5]. Increasing evidence has emphasized the importance of potential targets that can cooperate with ATRA treatment. For instance, a recent phase I/II clinical trial with ATRA plus a small-molecule inhibitor targeting lysine-specific demethylase 1 has achieved a significant clinical response in refractory or relapsed AML patients [6]. In the present study, we aimed to explore candidate targets for combination therapy with ATRA by establishing an ATRA-induced translatome atlas in AML cells.

Energetically, protein synthesis needs to be strictly controlled because of its high cost [7]. Mounting evidence has suggested that regulation of gene expression occurs not only at the transcriptional level but also at the translational level. Gene mutation as a consequence of transcriptional regulation may be offset by translational regulation. A study indirectly inferring translation efficiency from absolute mRNA and protein abundance measurements found that translation was the largest contributor to predicting protein abundance, highlighting the value of direct measurements of protein synthesis [8]. The classic translation process in eukaryotes is mainly divided into four steps: initiation, elongation, termination, and the release of the free polypeptide [9]. As studied in our previous research, several eukaryotic translation initiation factors (eIFs) were implicated in ATRA-induced leukemia cell development [10,11]. The 5′m7GpppN cap (cap)-dependent translation initiation is the classic translation initiation pattern in eukaryotes, accounting for most protein synthesis under normal growth conditions, and was dysregulated in many solid tumors and leukemia [12,13,14]. Regulated eIF4F complex assembly is an essential step in controlling cap-dependent translation initiation. During the remission of different types of leukemia induced by chemotherapy, plenty of factors related to proliferation, apoptosis, and differentiation were involved. Further studies of translation regulation in these factors may help to explore novel drug targets or enable AML remission.

In this study, ATRA and 4-Amino-2-Trifluoromethyl-Phenyl Retinate (ATPR), a novel ATRA derivative with substantial pharmacological activity on the differentiation of AML cells, both exerted significant inhibition in global translation and peptide synthesis. Further studies confirmed that the decrease in eIF4E, which is responsible for cap-dependent translation initiation, was critical for maintaining the effect of ATRA and ATPR on cellular proliferation and differentiation in AML cells. Subsequently, we performed a detailed assessment of the genome-wide translatomes in ATRA-treated cultured AML cells using ribosome profiling (Ribo-seq) [15] that might serve as the origin for characterizing the translational landscape of AML cells in response to ATRA stress. Aberrant activation of the PI3K/AKT signaling pathway at the translational level in response to ATRA or ATPR was revealed, and a sequential strategy including ATRA or ATPR plus a PI3K/AKT inhibitor was suggested as a potential treatment for AML.

## 2. Materials and Methods

### 2.1. Cell Lines and Cell Culture

Molm13 cell line was obtained from Beyotime Biotechnology (Shanghai, China). NB4 cell line was purchased from Genechem (Shanghai, China). MV4-11 cell line was obtained from Procell Life Science & Technology (Wuhan, China). HL60 and THP-1 cell lines were obtained from Gecko gene (Shanghai, China). Molm13, NB4, HL60, and THP-1 were maintained in regular RPMI-1640 medium (HyClone, Logan, UT, USA) supplemented with 10% FBS (Biological Industries, Beit Haemek, Israel) and 1% penicillin-streptomycin (Beyotime Biotechnology, Shanghai, China). MV4-11 was cultured in Iscove’s Modified Dulbecco’s medium (IMDM) (HyClone, Utah, USA), supplemented with 10% FBS and 1% penicillin-streptomycin. All cell lines were maintained at 37 °C in a 5% CO_2_ environment, and have passaged for fewer than 6 months in our laboratory after resuscitation.

### 2.2. Xenotransplantation Experiments

All animal experiments were performed in accordance with the institutional ethical guidelines for animal experiments (Ethics Number: LISC20190751). NOD-PrkdcscidIl2rgem1/Smoc (M-NSG) immunodeficient mice (Animal certificate number: 20170010009479) were obtained from the Shanghai Model Organisms Center (Shanghai, China). Male mice (6–9 weeks old) were housed and bred in the Laboratory Animal Center under a specific pathogen-free (SPF) facility (Anhui Medical University, Hefei, Anhui, China). For the subcutaneous tumor xenografts study procedure, 100 μL Molm13 or NB4 cells diluted in phosphate buffer saline (PBS) (HyClone, Logan, UT, USA) (5 × 10^7^ cells/mL) were subcutaneously injected in the flank of M-NSG mice. On day 14, all mice were sacrificed, and their tumors were stripped, weighed, and processed for protein extraction.

### 2.3. Human Samples and Cell Isolation

CD34^+^ hematopoietic stem/progenitor cells (HSPCs) were sorted from the cord blood of healthy donors from the First Affiliated Hospital of Anhui Medical University using Ficoll-Paque PLUS (GE Healthcare Life Sciences, Stockholm, Sweden) and anti-CD34-coated magnetic beads (Miltenyi Biotec, Bergishgradbach, Germany). Mononuclear cells were isolated from fresh cord blood as previously described. A single-cell suspension was incubated with anti-CD34-coated beads and selected on MACS (Miltenyi Biotec, Bergishgradbach, Germany). The purity of CD34^+^ cells was analyzed by flow cytometry (CytoFLEX, Beckman Coulter, Miami, FL, USA) following incubation with anti-CD34 antibodies (BioLegend, San Diego, CA, USA). Primary CD34^+^ HSPCs were cultured in StemSpan SFEM medium (StemCell Technologies, Vancouver, BC, Canada) supplemented with 2 mmol/L L-glutamine (Sigma-Aldrich, St. Louis, MO, USA), 1% Lipid Mixture 1 (L0288, Sigma-Aldrich, St. Louis, MO, USA), 100 ng/mL SCF (Human origin, PeproTech, Rocky Hill, CT, USA), 2 ng/mL IL-3 (Human origin, PeproTech, Rocky Hill, CT, USA), and 1% penicillin-streptomycin.

### 2.4. Preparation of Samples for Ribosome Profiling (Ribo-Seq)

Molm13 cells were treated with ATRA (10 μM, 72 h). The cells were washed twice with cooling PBS supplemented with cycloheximide (100 μg/mL) (MCE) and then snap-frozen in liquid nitrogen until lyse. To extract ribosome-protected RNA fragments (RPFs), cells were lysed and then released into RNase to digest free RNA. RPFs between 28–30 nt were recycled and enriched. After removing protein and rRNA, the library was prepared, high-throughput sequenced, and they were applied to analysis combined with RNA-seq data.

### 2.5. Analysis of Sequencing Data

First, the quality control of raw data was initially processed using FastQC, and adapter sequences and poor-quality reads were removed. Then, quality-filtered reads were mapped to the reference genome using Bowtie. After removing the mycoplasma contamination and ribosomal reads, quantitative analysis of Ribo-seq, distribution of uORFs, and sequence analysis of motifs were carried out. The translation level of the indicated gene was measured by translation efficiency (TE).

### 2.6. Cell Transfection

eIF4E silence or overexpression was achieved through lentiviral infection of shRNA targeting eIF4E or eIF4E-overexpression plasmids (Hanbio, Shanghai, China), and infections were performed according to the manufacturer’s manual. Stably infected cells were selected by puromycin. The selected cells were cultured in a medium containing puromycin. qPCR and western blot were employed to confirm the silence or enhancement of eIF4E in AML cells.

### 2.7. Protein Extraction and Western Blotting

Cells were collected, washed with cold PBS, and lysed with RIPA buffer (Beyotime Biotechnology, Shanghai, China) supplemented with PMSF (Beyotime Biotechnology, Shanghai, China). After centrifugation at 12,000 rpm for 30 min, the supernatant was transferred to a new tube, mixed with SDS-PAGE loading buffer, denatured by heat at 100 °C, and fractionated by SDS-PAGE. Then, the separated protein was transferred onto methanol-activated PVDF membranes (Millipore, Boston, MA, USA). After being blocked with 5% defatted milk for 2 h at room temperature, the membranes were then incubated with the appropriate diluted antibodies against eIF4E, eIF4A, eIF4G, eIF4EBP-1, p-Ser65-eIF4EBP-1, p-Thr37/46-eIF4EBP-1 (1:1 000, Cell signaling technology, Boston, MA, USA), CDK2, PCNA, AKT, p-ser473-AKT, Histone 3 (1:1000, Abcam, Cambridge, UK), β-actin (1:1000, Proteintech, Chicago, IL, USA), respectively, at 4 °C overnight. After the incubation, membranes were hatched with HRP-conjugated secondary antibody (1:8000, ZSGB-Bio, Beijing, China). The blots were visualized with Omni-ECL™Femto Light Chemiluminescence Kit (EpiZyme, Shanghai, China) and quantified with ImageJ software.

### 2.8. Puromycin Incorporation Assay

The global translation was measured by the incorporation of puromycin into the nascent peptides. 30 min before harvesting the cells, puromycin was added into the indicated culture medium at a final concentration of 10 μg/mL. The cells were then collected and applied to extract the total protein. The amount of puromycin incorporation was visualized with α-puromycin antibodies and determined by western blot.

### 2.9. Total RNA Extraction and Quantitative Real-Time PCR Analysis (qPCR)

Total RNA was extracted from cells using the Trizol procedure (Invitrogen, Carlsbad, CA, USA). RNA was reverse transcribed into cDNA with the reverse transcription system (Accurate Biology, Guangzhou, China). All primers were synthesized by Sangon Biotech (Shanghai, China) or General BIOL (Anhui, China). All the data were normalized to β-actin as an internal control.

### 2.10. Flow Cytometry for Cell Differentiation Analysis

The proportion of monocytes/granulocytes (CD11b-positive) and HSPCs (CD34-positive) were measured by flow cytometry. In brief, cells with different treatments were collected, washed with ice-cold PBS, and incubated with the specific antibodies with fluorescence labeling of differentiation markers, CD11b-PE (BioLegend, San Diego, CA, USA), or HSPCs marker, CD34-PE (BioLegend, San Diego, CA, USA) for 30 min in the dark at room temperature. Finally, the cells were washed twice with PBS and then analyzed on flow cytometry (CytoFLEX, Beckman Coulter, Miami, FL, USA).

### 2.11. Morphological Assessment

Cell morphology was illustrated by Wright-Giemsa staining, which stained the cytoplasm into blue and the nucleus into purple. In short, the cells were harvested, resuspended with 50 μL PBS, then uniformly coated on a glass slide. After being air dried, the cells were stained with Wright-Giemsa stain solution A for 1 min, B for 5 min, and rinsed with running water for 30 s. Finally, the slides were observed under a Fluorescence Inversion Microscope System (OLYMPUS, Tokyo, Japan).

### 2.12. Immunofluorescence Staining

After the indicated treatment, the cells were collected, washed with PBS twice, and then fixed with 4% paraformaldehyde for 60 min. 1% Triton X-100 in PBS was added to increase the permeability of the cell membrane. After washing with PBS twice, the cells were blocked with 5% *w*/*v* BSA/PBS at room temperature for 60 min. They were then incubated with primary antibodies against eIF4E or Ki67 diluted in 5% *w*/*v* BSA/PBS (1:200) overnight at 4 °C, and for 60 min at room temperature. Subsequently, the cells were washed twice with PBS and incubated with fluorescent-labeled secondary antibodies diluted in 5% *w*/*v* BSA/PBS (1:200) in the dark for 60 min. Finally, after being stained with DAPI for 5 min, cells were observed under a Fluorescence Inversion Microscope System (OLYMPUS, Tokyo, Japan) to detect the fluorescence intensity.

### 2.13. Cell Counting Kit-8 (CCK-8) Assay

The cell proliferation/growth was detected by CCK-8 assay using a CCK-8 kit (Topscience, Shanghai, China). The cells were seeded into 96-well plates and cultured for the indicated treatment. 10 μL CCK-8 was added into each well and mixed with the cells. After 2-h incubation, the OD value at 450 nm was detected by a microplate reader.

### 2.14. Colony Formation Unit (CFU) Assay

The cell colony-forming ability was assessed by the CFU assay. The semi-solid medium was formulated according to the manual provided by the manufacturer using MethoCult H410 and IMDM medium supplemental with 20% FBS. Cells from the indicated treatments were collected, washed with PBS once, then diluted to a concentration of 2 × 10^6^/mL. 10 μL cell dilution was mixed with 1.5 mL semi-solid medium (avoiding bubbles), and plated in a 6-well plate. After incubation for two weeks in a humidified atmosphere with 5% CO_2_ at 37 °C, the cell clone was imaged under a Fluorescence Inversion Microscope System (OLYMPUS, Tokyo, Japan) and analyzed by ImageJ.

### 2.15. Apoptosis by Flow Cytometry

The cell apoptosis was measured using Annexin V-FITC/propidium iodide (PI) double-staining assay (Bestbio, Shanghai, China) and detected by flow cytometry. The analytical procedures were performed in accordance with the manual provided by the manufacturer.

### 2.16. TdT-Mediated dUTP Nick-End Labeling (TUNEL) Assay

The cell apoptosis was detected by TUNEL assay using a one-step TUNEL Apoptosis Detection Kit (Beyotime, Shanghai, China). The cells were collected, washed with PBS, and fixed with 4% paraformaldehyde for 30 min. After washing with PBS once, the cells were permeated with 0.3% Triton X-100 for 5 min. They were then incubated with 50 μL TUNEL staining consisting of 5 μL TdT and 45 μL fluorescent labeling solution for 60 min in the dark at 37 °C. Finally, the cells were washed with PBS twice, and resuspended with 500 μL PBS, and the fluorescence intensity was analyzed by flow cytometry.

### 2.17. Statistical Analysis

Differences in data with normal distribution were analyzed by Student’s *t*-test. Non-normally distributed data were analyzed by Mann-Whitney nonparametric test. Error bars depict mean ± SEM. ns: no significance, * *p* < 0.05; ** *p* < 0.01; *** *p* < 0.001.

## 3. Results

### 3.1. ATRA and ATPR Inhibit Global Translation and Peptide Synthesis

**Figure 1:**
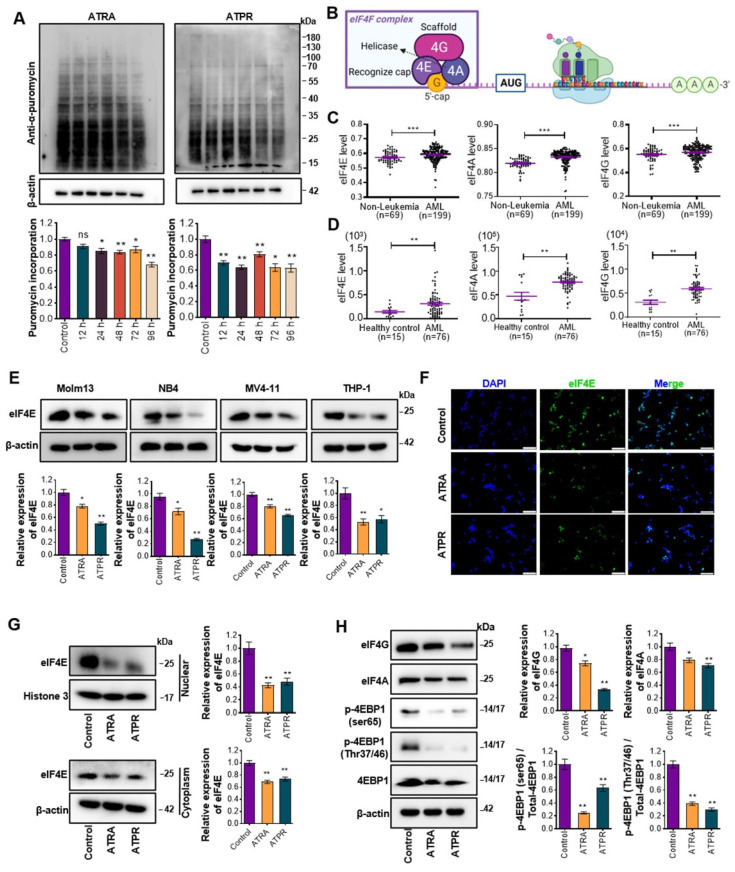
ATRA and ATPR inhibited global translation and protein synthesis. (**A**) Global nascent peptide synthesis was measured by puromycin incorporation assay and detected using western blot with anti-α-puromycin antibodies. (**B**) Schematic representation of the eIF4F complex during translation. (**C**,**D**) The expression level of eIF4F complex components eIF4E, eIF4A, and eIF4G in AML patients compared with healthy controls or patients without leukemia were analyzed via retrieving the microarray data in the Gene Expression Omnibus (GEO) datasets (C, GSE15061; D, GSE12662). (**E**) Protein expression of eIF4E in AML cell lines Molm13, NB4, MV4-11, and THP-1 with ATRA or ATPR exposure were analyzed by western blot. (**F**) Immunofluorescent staining of Molm13 cells showing expression of eIF4E (Green) with DAPI (Blue); bar, 100 μm. (**G**) Western blot analysis showing the distribution of eIF4E in cytoplasmic and nuclear of Molm13 cells with ATRA or ATPR exposure (10 μM, 72 h). (**H**) Western blot analysis showing protein level of eIF4G, eIF4A, eIF4EBP1 (4EBP1), and phosphorylation level of 4EBP1 at ser65 and Thr37/46 in Molm13 cells during ATRA or ATPR stress. β-actin served as a loading control. ns: no significance, * *p* < 0.05; ** *p* < 0.01; *** *p* < 0.001.

FLT3 mutations are the most common genetic abnormality in patients with AML, with ITD accounting for approximately 25% of all AML cases, and a tendency to adverse clinical outcomes [16]. As a model, we selected the AML cell line Molm13 which carries FMS-like tyrosine kinase 3-internal tandem duplication (FLT3-ITD) to analyze the contribution of ATRA or ATPR-dependent translation to AML, for its sensitivity to ATRA and ATPR in our previous study. 72 h of exposure of Molm13 cells to ATRA or ATPR promoted cell differentiation by ~54% or ~39%, respectively (ATRA, Figure S1A; ATPR, Figure S1B). Likewise, the effect of ATRA and ATPR on global translation was evaluated by visualizing puromycin incorporation into newly synthesized polypeptides and coomassie brilliant blue staining of total protein. A concentration of 10 μM ATRA or ATPR was selected, which exerted inhibition on global translation (decline in de novo protein and reduced total protein content) after 72 h of exposure in Molm13 cells (Figure 1A and Figure S2A). Translation initiation is the rate-limiting step and is responsible for the post-transcriptional regulatory diversity [17]. We surmised that the cap-dependent translation initiation complex eIF4F (Figure 1B) was involved in ATRA or ATPR-regulated AML development. Analysis of the GSE15061 (Figure 1C) and GSE12662 (Figure 1D) datasets downloaded from the Gene Expression Omnibus database revealed that eIF4E, eIF4A, and eIF4G were both abnormally higher expressed in AML patients compared with non-leukemic patients or healthy control. Our results showed that ATRA and ATPR decreased the protein level of eIF4E in the four AML cell lines (NB4, Molm13, MV4-11, and THP-1) (Figure 1E), and subsequent results found in the immunofluorescence staining assay also confirmed that ATRA and ATPR inhibited endogenous eIF4E levels (Figure 1F). eIF4E resides in nuclear and cytoplasm where it plays distinct roles in the export and translation of target transcripts, respectively [18,19]. Following cytoplasmic and nuclear fractionation-western blots assays, we next uncovered that ATRA and ATPR caused a reduction in both nuclear and cytoplasmic eIF4E (Figure 1G). Furthermore, an inhibitory effect of ATRA and ATPR on the protein level of eIF4G and eIF4A was also observed in Molm13 (Figure 1H). During eIF4F complex loading, eIF4E might undergo a translational repress by eIF4E-binding proteins (4E-BPs), which share the same eIF4E-binding motif with eIF4G and thus inhibits the formation of the eIF4F complex [20]. Significantly, ATRA and ATPR decreased phosphorylation of the eIF4E-binding protein 1 (4EBP1) at Ser65 and Thr37/46 sites and led to the anchor of 4EBP1 on eIF4E, therefore impeding the binding between eIF4E and eIF4G (Figure 1H). Additionally, the same effects of ATRA and ATPR on eIF4E, eIF4G, and eIF4A were observed in another AML cell line NB4, originating from acute promyelocytic leukemia (APL) patient with high-sensitive to ATRA-induced cell differentiation (Figure S2B–D). These results suggested that ATRA and ATPR inhibited global translation and peptide synthesis in AML.

### 3.2. Decrease in the Translation Initiator eIF4E was Critical for Maintaining the Efficiency of ATRA and ATPR in AML

**Figure 2:**
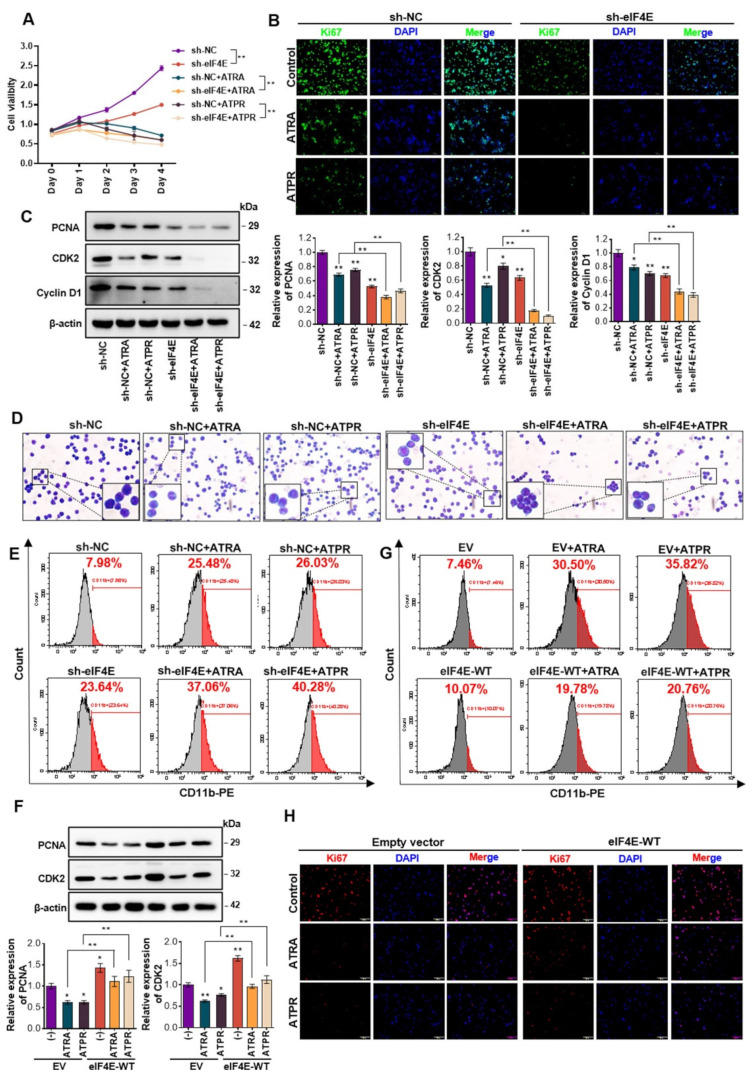
eIF4E was a critical regulator in ATRA or ATPR-induced AML cell growth arrest and differentiation. (**A**) Molm13 cells were infected with lentiviral-based shRNA targeting eIF4E or a negative control scramble shRNA and then exposed to ATRA or ATPR. Cell growth/proliferation was evaluated by cell counting kit-8 (CCK-8). (**B**) Immunofluorescent staining showing proliferating Molm13 cells by labeling endogenous Ki67 (Green) with DAPI (Blue); bar, 100 μm. (**C**) Western blot analysis and quantification of proliferating cell nuclear antigen PCNA and cell cycle regulators CDK2 and CyclinD1. (**D**) Wright-Giemsa staining showing the morphological maturity of Molm13 cells after specific treatments. Purple: Nuclear. Blue: Cytoplasm. Bar, 50 μm. (**E**) The proportion of cell differentiation marker CD11b-positive Molm13 cells was analyzed by flow cytometry. (**F**) eIF4E was overexpressed in Molm13 cells by lentiviral-based eIF4E-WT or negative control scramble plasmids infection, and then cells were treated with ATRA or ATPR. Cell proliferation was assessed by protein levels of PCNA and CDK2 and detected by Western blot. (**G**) Cell differentiation was analyzed by the proportion of CD11b-positive cells and detected by flow cytometry. (**H**) Immunofluorescent staining of Ki67 (Red) with DAPI (Blue) in Molm13 cells from the indicated groups; bar, 100 μm. * *p* < 0.05, ** *p* < 0.01.

Given that the specific recognition of mRNA 5′cap by eIF4E is a rate-limiting step in translation initiation, we have implicated the eIF4F complex, especially the eIF4E component, as an important regulator in AML cells. We next assessed whether eIF4E plays a role in AML cells in the absence or presence of ATRA and ATPR exposure. eIF4E was knocked down using lentivirus-based shRNA targeting eIF4E in Molm13 and NB4 cells, and the knockdown efficiency was determined in mRNA (Figure S3A) and protein level (Figure S3B). Following genetic knockdown of eIF4E, both of the two AML cell lines had reduced viability when assessed by CCK-8 assays (Molm13, Figure 2A; NB4, Figure S3C) and subsequently inhibited proliferation when assessed by Ki67 labeling fluorescence intensity (Molm13, Figure 2B; NB4, Figure S3D). Mechanically, the expression of specific proteins that regulate the G0/G1 cell cycle, the cyclinD1, the cyclin-dependent kinase 2 (CDK2), and the proliferating cell nuclear antigen (PCNA) were all downregulated by eIF4E knockdown (Molm13, Figure 2C; NB4, Figure S3E). These results suggested that a high level of eIF4E maintained aberrant proliferation in AML cells. Furthermore, a synergistic effect of eIF4E depletion and ATRA or ATPR on the inhibition of cell growth/proliferation was observed, characterized by a further reduction in cell viability, Ki67-labeled fluorescence intensity, and expression of PCNA, cyclinD1, and CDK2. Subsequently, we showed that eIF4E knockdown promoted the process of terminal differentiation in AML cells. As seen in the enhanced proportion of myeloid differentiation surface marker CD11b-positive cells, eIF4E knockdown rescued cell differentiation in the absence of ATRA or ATPR (Molm13, Figure 2E; NB4, Figure S3F). In addition, the morphological changes of AML cells were illustrated when stained with Wright-Giemsa, and cells with knockdown of eIF4E showed an indentation and bending of the nuclei and a decrease in nuclear/cytoplasmic ratio, suggesting a terminally differentiated tendency in eIF4E-depleted cells (Molm13, Figure 2D; NB4, Figure S3G). Consistent with the effect of eIF4E on cell proliferation, we found that silencing eIF4E also enhanced the capacity of ATRA or ATPR to induce AML cell differentiation, characterized by a further elevation in CD11b-positive cell ratio and morphological maturity. That is, AML cells with impaired eIF4E expression were more sensitive to ATRA- and ATPR-induced cell growth arrest and differentiation.

Then, whether sustained eIF4E disturbed the regulation of cell proliferation and differentiation induced by ATRA or ATPR needs to be determined. To this end, we enforced eIF4E expression in AML cells by infecting cells with lentivirus-based eIF4E-WT plasmids and confirmed the overexpression efficiency at the mRNA (Figure S3H) and protein levels (Figure S3I). eIF4E-overexpressing cells were then subjected to ATRA or ATPR exposure. eIF4E-overexpressing AML cells were more aggressive with a high level of PCNA, CDK2 (Figure 2F), and Ki67 (Figure 2H). As expected, the downregulation of PCNA, CDK2, and Ki67 induced by ATRA or ATPR was significantly counteracted by eIF4E. Besides, we noted that eIF4E impeded the process of AML cell maturation, as sustained eIF4E inhibited the induction of CD11b expression by ATRA or ATPR (Figure 2G), suggesting that the inhibition of eIF4E was critical to maintaining the effect of ATRA or ATPR on AML cell differentiation. Collectively, we suggested that eIF4E, the crucial regulator in cap-dependent translation, plays an important role in AML progression and the sensitivity of AML cells to ATRA or ATPR.

### 3.3. eIF4E Knockdown Prevents the Onset and Progression of AML Tumorigenesis In Vivo

**Figure 3:**
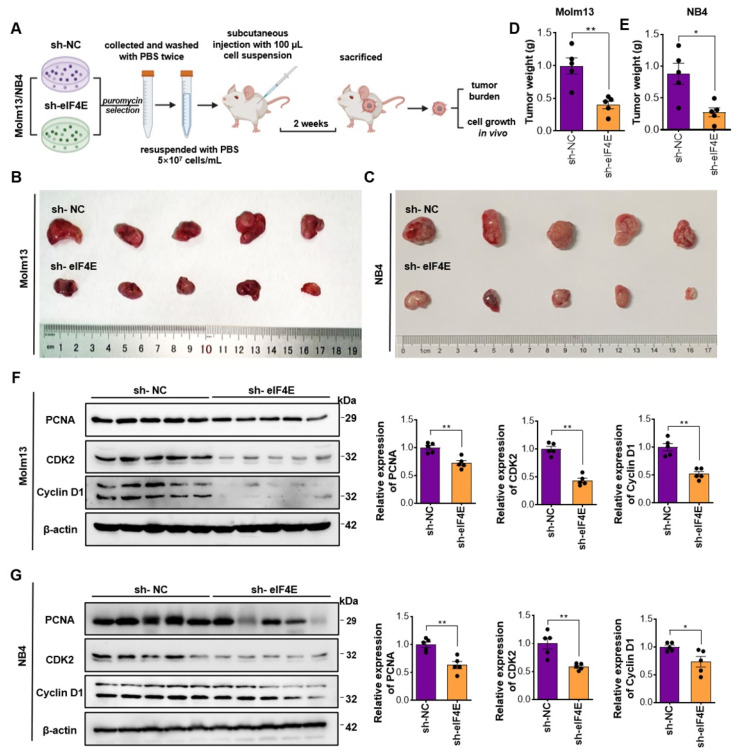
eIF4E knockdown blocked the AML cell growth in vivo. (**A**) Schematic representation of in vivo AML xenograft experiment. M-NSG mice received subcutaneous injection with 5 × 10^6^ indicated Molm13 cells. (**B**,**C**) Two weeks after the injection, the mice were sacrificed and the tumors were removed and photographed. (**D**,**E**) AML xenograft tumor burden was analyzed by weighting the tumors. (**F**,**G**) The cell proliferation of AML cells in vivo was assessed by the protein level of PCNA, CDK2, and CyclinD1. Total proteins were extracted from tumor tissues and subjected to western blot. * *p* < 0.05, ** *p* < 0.01.

The above results supported the therapeutic potential of eIF4E in AML in the absence of ATRA or ATPR in vitro, we then aimed to determine the role of eIF4E in vivo by establishing an AML xenograft model (Figure 3A). Molm13 or NB4 cells were infected with lentivirus-based shRNA targeting eIF4E or negative control (NC), then subjected to puromycin selection to establish the indicated stable cell line. M-NSG, severely immunodeficient M-NSG mice received a subcutaneous injection with 5 × 10^6^ eIF4E-kncokdown or NC cells in the flank. Two weeks after injection, tumor burden and cell proliferation in vivo were assessed. We found that the tumorigenesis of the eIF4E-knockdown Molm13 and NB4 cells was impaired, as shown by a significant decrease in tumor size (Figure 3B,C) and weight (Figure 3D,E). Western blot using protein extracted from AML tumors suggested that eIF4E knockdown inhibited the expression of PCNA, CDK2, and CyclinD1, demonstrating the reduced AML cell proliferation in vivo (Figure 3F,G). Data support the strategy targeting eIF4E in AML therapy.

### 3.4. ATRA Reprograms Translation in AML Cells

**Figure 4:**
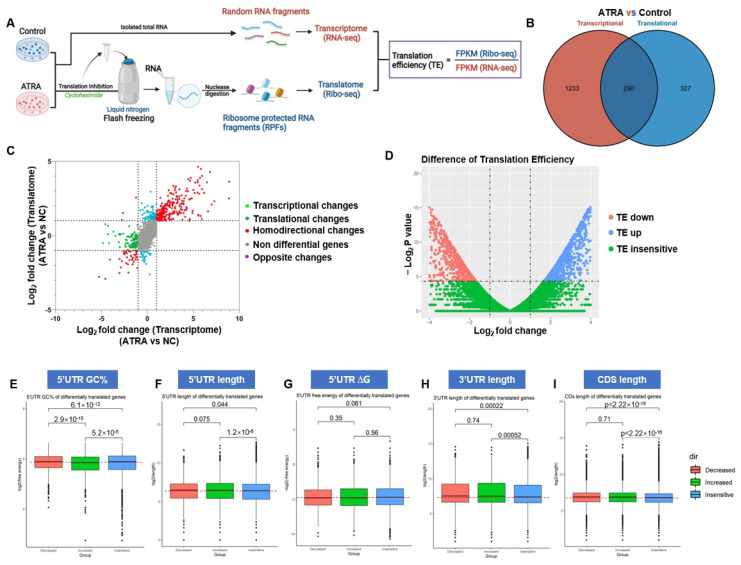
Translation atlas in AML cells in response to ATRA stress was established using RNA-seq combined with Ribo-seq. (**A**) Schematic representation of the sequencing flow chart and definition of TE for each gene. (**B**) Identification of genes significantly regulated by ATRA using RNA-seq (transcriptome) and Rbio-seq (translatome). The number of genes transcriptionally or translationally regulated by ATRA is shown. (**C**) Nine quadrant diagram showing the distribution of genes in transcriptome and translatome. (**D**) Volcano plot of genes with different TE; TE, translational efficiency. (**E**–**G**) GC content (**E**), length (**F**), and free energy (**G**) of 5′UTR of differentially translated genes during ATRA stress. (**H**,**I**) Length of 3’UTR (**H**) or CDs (**I**) of differentially translated genes during ATRA stress.

To further examine the effect of ATRA on translation, we employed ribosome profiling (Ribo-seq) combined with transcriptome sequencing (RNA-seq) to perform a detailed characterization of the translatome and transcriptome of cells in response to ATRA (Figure 4A). The Ribo-seq technique allows for in-depth quantified ribosome-protected mRNA fragments (RPFs). We treated Molm13 cells with 10μM ATRA for 72 h, then deep-sequenced RPFs and total RNA. For bioinformatics analyses, raw sequence data were initially processed by FastQC for quality control, and then adapter sequences and poor-quality reads were removed. The data from Ribo-seq showed that the length of the sequenced fragments in all samples ranged from 26 to 32 nucleotides, the expected size distribution of RPFs. By conducting matched RNA-seq data and Ribo-seq data, we contrasted genome-wide transcriptional and translational differences. Notably, 327 translatome-only differences were found in ATRA samples comparing control samples (Figure 4B,C). To make the gene translation level of different genes and different experiments comparable, we introduced a measure to define the number of fragments from a gene per kilobase length per million reads (FPKM). The translational efficiency (TE) value for each transcript was calculated by normalizing the FPKM value of RPFs from Ribo-seq data to the FPKM value of transcript from RNA-seq data, that is, TE = FPKM (Ribo-seq)/FPKM (RNA-seq), thereby revealing the transcript-specific utilization by translation component (Figure 3A). The fold change in TE (ΔTE) was calculated by dividing TE values from ATRA-treated cells by those from control cells, thus ∆TE = TE (ATRA)/TE (control) for each gene (Figure 4D). The ΔTE value provides a quantitative measure of the translation regulation of ATRA. We identified groups of genes whose TE was downregulated (TE down; red) or upregulated (TE up; blue) affected by ATRA compared to the background (TE insensitive; green). The TE down group includes 1324 genes, and the TE up group includes 1664 genes. Gene ontology (GO) analysis of TE-sensitive (including TE down and TE up) genes indicated that a lot of biological processes or cellular components, including calcium ion binding, transmembrane receptor protein tyrosine kinase signaling pathway, cell morphogenesis involved in differentiation, and ion channel complex, were perturbed by ATRA, revealing that ATRA translationally regulated AML cell development (Figure S4).

Although genetic information is transmitted from DNA to mRNA coding sequences (CDs), the role of 5′ and 3′ untranslated regions (UTRs) are particularly important in modulating gene translation. To further characterize the transcripts that are translationally regulated by ATRA, we focused our research on the secondary structure of 5′UTRs and 3′UTRs. High GC content (GC%), overall low free energy (ΔG), and the length of a 5′UTR are often used as parameters for predicting 5′UTR RNA secondary structure. We compared the ATRA-sensitive and ATRA-insensitive groups and confirmed that the pool of 5′UTRs from TE down genes in the ATRA-sensitive group was enriched with a complex secondary structure. The difference in GC% between genes with decreased TE in ATRA and insensitive genes was highly significant (*p* = 6.1 × 10^−12^) and a difference in the distribution of GC% from genes with increased TE was also notable compared with the insensitive pool (*p* = 5.2 × 10^−5^; Figure 4E). Besides, the TE of genes with low GC% 5′UTR was increased in the TE-sensitive group, suggesting that mRNA with simple secondary structure 5′UTR had a translation advantage in AML cells during ATRA stress. Evaluation of 5′UTRs for length revealed that the translation of mRNA with longer 5′UTR was significantly disturbed by ATRA (Figure 4F). As 5′UTR provides a platform for interaction with regulators, longer 5′UTR leads to increased accessibility of translation regulation. In our research, TE up and TE down mRNAs both had longer 5′UTR than those from insensitive mRNAs, suggesting that: (a) long 5′UTR in the sensitive genes allowed different elements to play specific roles in mRNA translation; and (b) the detailed range of 5′UTR lengths might be needed to presume regulation of specific mRNA subsets. In addition, the relevance of the stability of RNA structures and translation has not been established, as we found no significant difference for ΔG value in ATRA-sensitive and -insensitive genes (Figure 4G). These data suggested that ATRA-sensitive genes have a 5′UTR signature characterized by long sequences with complex secondary elements. Since 5′UTR of ATRA-sensitive genes would be more structured than genes insensitive to ATRA, we noted the important roles of eIF4F, especially eIF4E, in ATRA-induced translational regulation. Likewise, we confirmed that lengths of 3′UTR or CDs in the ATRA-sensitive pool were also longer than those from insensitive genes (3′UTR, Figure 4H; CDs, Figure 4I), again indicating that the function of ATRA in regulating gene translation could have specific effects on target mRNAs, at least in part through abundant RNA elements in UTRs or CDs.

### 3.5. PI3K/AKT Signaling Activation Was Required for ATRA- or ATPR-Induced AML Cell Differentiation

**Figure 5:**
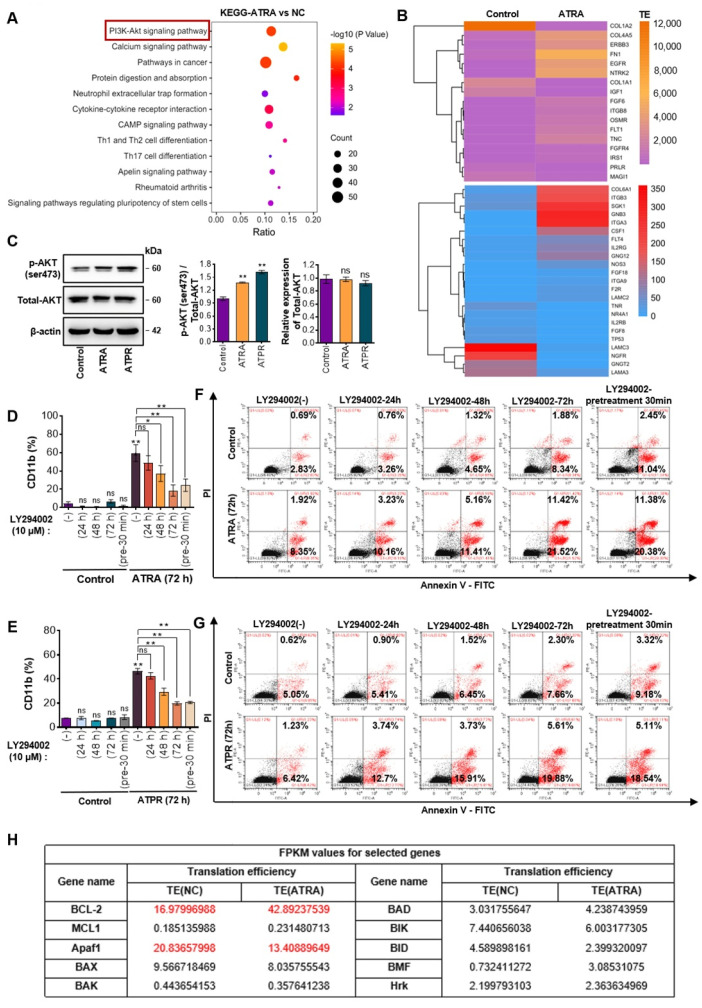
PI3K/AKT signaling was activated by ATRA and ATPR. (**A**) KEGG enrichment analysis of TE-sensitive genes (including TE down-regulated genes and TE up-regulated genes) during ATRA stress. (**B**) Heatmap of TE value for TE-sensitive genes enriched in the PI3K/AKT pathway. (**C**) PI3K/AKT activity was evaluated by detecting the protein expression of AKT and the phosphorylation level of AKT at ser473 using western blot. (**D**,**E**) Molm13 cells were simultaneously treated with ATRA (10 μM, (**D**)) or ATPR (10 μM, (**E**)) and LY294002 (10 μM), a PI3K/AKT inhibitor, for the indicated times. Cell differentiation was analyzed by quantifying the proportion of CD11b-positive cells by flow cytometry. (**F**,**G**) Apoptosis of cells treated with ATRA (**F**) or ATPR (**G**) and LY294002 was assessed by flow cytometry using an Annexin V-FITC/PI staining kit. In the “LY294002-pretreatment 30 min” panel, cells were pre-treated with PI3K/AKT inhibitor (LY294002) for 30 min, and then ATRA or ATPR were directly added to the medium containing LY294002. (**H**) Fragments per kilobase of exon per million fragments (FPKM) values of apoptotic genes translationally regulated by ATRA. ns, not significant; * *p* < 0.05, ** *p* < 0.01.

As we have established the translation atlas in response to ATRA stress, we next carried out the Kyoto Encyclopedia of Genes and Genomes (KEGG) analysis to further explore the signal that was perturbed by ATRA. KEGG analysis of ATRA-sensitive genes revealed that many genes were enriched in the PI3K/AKT signaling pathway (Figure 5A). TE values of genes involved in PI3K/AKT signaling was shown in Figure 5B. We next focused on the role of PI3K/AKT in AML cells in the presence of ATRA or ATPR by monitoring cell differentiation and apoptosis. First, using western blotting, we confirmed that ATRA and ATPR strongly phosphorylated AKT at the serine273 site, which is necessary for AKT complete activation, whereas no significant changes were observed in the expression of AKT, suggesting that PI3K/AKT signaling was activated by ATRA or ATPR (Figure 5C). We then introduced LY294002 to antagonize the PI3K/AKT signaling in AML cells during ATRA or ATPR treatment. Inactivation of PI3K/AKT signaling caused a decrease in the fluorescence intensity of CD11b promoted by ATRA or ATPR, confirming the contribution of PI3K/AKT activation in ATRA or ATPR-induced cellular differentiation (ATRA, Figure 5D and Figure S5A; ATPR, Figure 5E and Figure S5B). Next, the effect of LY294002 on cell apoptosis was analyzed. Our results showed that the elimination of PI3K/AKT signaling slightly promoted cell apoptosis, whereas co-treatment with ATRA or ATPR enhanced the apoptosis in AML cells, albeit still slightly (ATRA, Figure 5F; ATPR, Figure 5G). These results suggested that activation of PI3K/AKT was required to preserve the activity of ATRA or ATPR in AML cells. However, activation of PI3K/AKT signaling was reported to trigger cell survival and drug resistance to ATRA in AML cells.

### 3.6. Sequential Strategy Combing PI3K/AKT Inhibitor Following ATRA or ATPR Eliminated AML Cells

**Figure 6:**
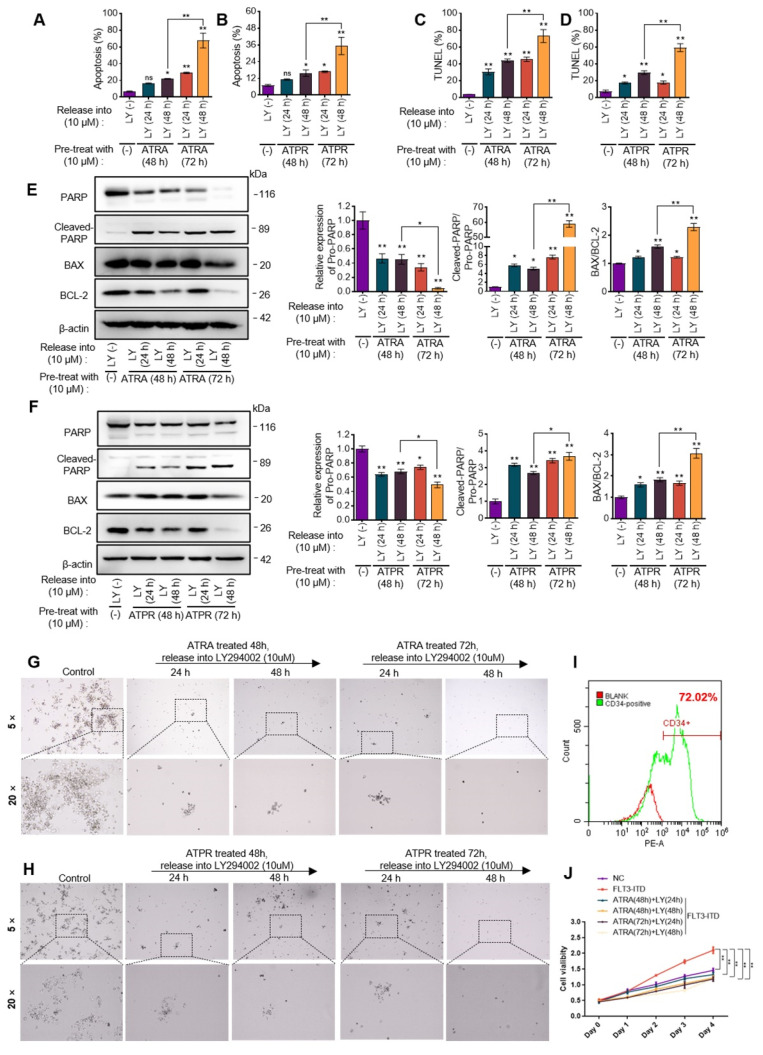
Sequential strategy combing PI3K/AKT inhibitor following ATRA or ATPR eliminated AML cells. The sequential strategy was that Molm13 cells were pretreated with ATRA or ATPR (10 μM) for 48 or 72 h and then released into LY294002 (10 μM) for 24 or 48 h. Then, (**A**,**B**) Cell apoptosis were detected by flow cytometry using an Annexin V-FITC/PI staining kit ((**A**) ATRA; (**B**) ATPR). (**C**,**D**) TUNEL analysis was processed to further measure the degree of cell apoptosis, and flow cytometry was utilized to quantify the cell apoptosis ((**C**) ATRA; (**D**) ATPR). (**E**,**F**) Western blot showing the protein level of anti-apoptotic BCL-2, pro-apoptotic BAX, PARP, and its cleavage ((**E**) ATRA; (**F**) ATPR). The ratio of BAX relative to BCL-2 and cleaved-PARP relative to pro-PARP were used to assess cell apoptosis. (**G**,**H**) The self-renewal capacity of Molm13 cells was analyzed by cell clonality and illustrated by colony forming unit (CFU) assay ((**G**) ATRA; (**H**) ATPR). (**I**) The sorting efficiency of the CD34+ HSPCs was assessed by the proportion of CD34-positive cells and detected by flow cytometry. Blank, cells not incubated with CD34 antibody. (**J**) Effect of sequential strategy combing ATRA and LY294002 on FLT3-ITD-driven rapid cell growth/proliferation was measured using CCK-8. ns, not significant; * *p* < 0.05, ** *p* < 0.01.

Confirming the importance of PI3K/AKT, we subsequently determined whether the combination of PI3K/AKT inhibition and ATRA could exert synergy effects in clearing AML cells. Based on our Ribo-seq data, several apoptotic genes, such as BCL-2, MCL-1, and Apaf-1, were both translationally regulated by ATRA. BCL2 has been associated with decreased sensitivity of AML cells to cytotoxic chemotherapy and a higher rate of relapse due to its activity of protecting cells against apoptosis. However, the ∆TE data of BCL-2 suggested that the translation of the BCL-2 transcript was advanced by ATRA. On the contrary, protein expression of BAX, and Apaf-1, two pro-apoptotic genes, were translationally delayed in AML cells during ATRA stress, as shown by the negative ∆TE value (Figure 5H). These results indicated that AML cells might protect themselves by regulating apoptotic gene expression at the translation level, which might be implicated in the poor efficacy of ATRA in AML cells. However, previous regimens using PI3K/AKT inhibitor and ATRA simultaneously did not significantly induce apoptosis. Studies have reported that ATRA-induced differentiation sensitized AML cells to the induction of apoptosis. We then introduced sequential treatment including ATRA or ATPR and LY294002. We found that LY294002 treatment following ATRA or ATPR treatment induced a significant increase in cell apoptosis when assessed by Annexin-V/7AAD staining (Figure 6A,B, Figure S6A). TUNEL analysis verified the notable effects of the sequential strategy on the induction of cell apoptosis (Figure 6C,D, Figure S6B). Furthermore, western blotting showed that sequential treatment on AML cells could increase the cleaving of PARP and the ratio of BAX to BCL-2 (Figure 6E,F). The clinical success of differentiation therapeutic strategies depends on the irreversibility of LSCs differentiation; however, studies in AML or APL models have suggested that differentiated LSCs can revert into immature cells and reacquire leukemogenic properties. Thereby, loss of clonality is more suitable to serve as an endpoint of differentiation therapy [21,22]. These observations encouraged us to evaluate the cloning ability of AML cells after exposure to the sequential treatment by human colony-forming unit (CFU) assays. The results illustrated a notable inhibition of cloning ability in AML cells, and the clonal population of AML cells completely disappeared on a semi-solid medium (Figure 6G,H, Figure S6C,D), suggesting that PI3K/AKT inhibitors have the potential to display potent antileukemic activity against AML cells by enhancing the anti-cloning activity of ATRA monotherapy.

To determine the efficiency of the sequential treatments in eliminating AML cells in vivo, we next established the primary AML model by forcibly expressing FLT3-ITD in primary human HSPCs. Firstly, CD34+ HSPCs were sorted from human cord blood using anti-CD34-coated magnetic beads and MACS (Figure S6E). The results detected by flow cytometry confirmed the sorting efficiency (Figure 6I). Then the HSPCs were infected with lentivirus-based FLT3-ITD plasmids, allowing cell proliferation until we confirmed that FLT3-ITD transformed HSPCs, as evidenced by rapid cell growth. Subsequently, FLT3-ITD-HSPCs were exposed to ATRA and then released into LY294002 (Figure S6F). Results observed from the CCK-8 assay showed that the rapid cell proliferation of HSPCs driven by FLT3-ITD was significantly inhibited by the sequential treatment (Figure 6J).

Taken together, we suggested the sequential treatment combing ATRA and PI3K/AKT inhibitors as a potential therapy for AML.

## 4. Discussion

Early studies using ATRA as monotherapy in AML demonstrated only modest efficacy in AML patients, especially in patients with FLT3-ITD mutation [23]. Improving outcomes for patients with AML will require approaches involving novel drug combinations that overcome the disadvantages of ATRA. Based on that, mRNA translation was perturbated by ATRA and translation regulation by eIF4E was involved in ATRA activity in AML, we applied the concept of translationomics to establish the ATRA-induced specific translational landscape. We found that mRNAs involved in the PI3K/AKT signal were translationally impacted by ATRA stress, leading to the specific expression of genes that supported the activation of PI3K/AKT. This has been challenged by parabiosis studies that showed that the PI3K/AKT signal supported cell survival and promoted the stemness of AML cells [24,25]. The continuous activation of PI3K/AKT conferred by ATRA dramatically gave growth advantages to AML cells, which might be responsible for the failure of ATRA monotherapy in AML patients, as shown in our results that ATRA alone failed to induce obvious cell apoptosis in AML. We subsequently attempted to introduce PI3K/AKT inhibitors in the ATRA therapy strategy. Unexpectedly, the activation of PI3K/AKT was found to be required for ATRA to induce cell differentiation in AML cells, and PI3K/AKT activity elimination counteracted ATRA-induced AML cell differentiation without stimulating cell death, indicating that the simultaneous application of the PI3K/AKT inhibitor limited the maximal therapeutic effect of ATRA. In our previous research, the PI3K/AKT signal was transiently activated and eventually inhibited by ATPR in APL cells, suggesting the dynamic role of PI3K/AKT in APL cell differentiation [26]. In this regard, the sequential treatment was taken into account. Although combination regimens have become indispensable approaches for effectively treating cancer, the limitation of the traditional combination therapies by coordination issues that fail to account for the action sites of each drug should be taken into consideration [27]. A novel therapeutic approach using sequential treatment of ATRA and PI3K/AKT inhibitor remarkably induces cell apoptosis and suppresses cell clone formation in cultured AML cell lines. Importantly, the accelerated proliferation of CD34+ HSPCs driven by FLT3-ITD was also suppressed by the sequential treatment. Thus, it is becoming increasingly clear that further reversing the activation of PI3K/AKT following ATRA treatment enlarged the activity of ATRA in eliminating AML cells.

The most predominant cause of cancer treatment variation is drug resistance, and aberrant activation of PI3K/AKT seems to be responsible for adaptive resistance in several anti-cancer therapies. Similar to our study, cisplatin also upregulated PI3K/AKT activity, thus leading to a pro-survival signal in cancer cells, limiting its anticancer efficacy. The administration of sequential treatments of cisplatin and PI3K/AKT inhibitor could improve the poor efficacy, supporting the case that sequential treatment including PI3K/AKT inhibitor could be adopted for various anti-cancer strategies [28]. Similarly, one of the mechanisms underlying resistance to the anti-tumor antibiotic doxorubicin is the abnormal increase in the anti-apoptotic protein BCL-2. A study that sequentially delivered doxorubicin and Bcl-2-targeting siRNAs by respectively encapsulating each agent in tumor-homing nanoparticles found that the sequential treatments significantly achieved optimal efficacy in PC3-tumor-bearing mice [29]. In addition to avoiding conflicts between the molecular target of each drug, sequential treatments have advantages in preventing drug-drug interactions, preserving the pharmacokinetic and pharmacodynamic properties of either agent and minimizing the severe side effects caused by simultaneous co-delivery. Furthermore, differentiated cells were shown to be more sensitive to apoptosis induction, considering that the sequential strategy induced explosive AML cell apoptosis, this might be another advantage of the sequential strategy to enlarge the efficiency of ATRA [30]. Collectively, based on our results, we suggest that the sequence of administration of the drugs significantly impacted the anticancer outcome, and that the sequential treatment, including ATRA and PI3K/AKT inhibitor, exerts an optimal effect in AML, which needs further animal and clinical data. Besides, we advise that buparlisib, instead of LY294002 employed in our study, is more suitable for animal and clinical studies [31]. Buparlisib is a selective PI3K/AKT inhibitor, which has been used in clinical trials, such as in recurrent glioblastoma, advanced breast cancer, and metastatic squamous cell carcinoma, and has significant anti-tumor effects [32,33,34]. Further study can provide a theoretical basis for whether AML patients can benefit from buparlisib.

We described that the translational activity of mRNAs with complex and long 5′UTR was more sensitive to being perturbed by ATRA, and that transcripts with simple 5′UTR have translation advantages in ATRA stress. Many elements in 5′UTR have important roles in regulating translation, such as internal ribosome entry sites (IRES), RNA G-quadruplex (G4s), and pseudoknot, et al. [35]. IRES-dependent translation is a backup for most tumors to cope with their abundant needs during stress conditions where the cap-dependent translation is arrested, for instance, by mTOR inhibitors [36,37]. IRES-dependent translation of critical mRNA involved in cell cycle progression and anti-apoptosis maintained the tumor cell survival. Recent research has attributed the efficacy of a novel combination of pegylated crisantaspase and the BCL-2 inhibitor venetoclax in complex karyotype AML to global translation inhibition by enhancing 4EBP/eIF4E interaction on the cap-binding complex [38]. However, the translation of BCL-2 could be specifically compensated by IRES activation, as researchers have reported that IRES-dependent BCL-2 contributed to the malignant element of RPL10 R98S mutation in pediatric T-cell acute lymphoblastic leukemia [39]. When the global cap-dependent translation was rapidly inhibited, cancer cells would replenish the BCL-2 level by IRES to adapt to cell apoptosis stress. We presume that BCL-2 translation persisting in ATRA stress might also be dependent on IRES activation. Additional studies are warranted to further explore the roles of IRES in the translational activation of BCL-2 expression in ATRA-expose cells. Due to the important role of IRES activation in tumor cell survival, lots of researchers have developed anti-tumor compounds targeting IRES. A small molecule C11, which specifically inhibited the IRES activity of c-MYC by blocking the interaction of hnRNP A1 and IRES, exerted a synergistic anti-glioblastoma effect in combination with mTOR inhibitor rapamycin or PP242. Another study suggested that IRES-mediated translational activation is detrimental to cell differentiation and further reported that IRES small-molecule inhibitor cpd_*p* induced significant terminal differentiation in triple-negative breast cancer, ER-positive breast cancer, glioblastoma, and osteosarcoma cells, demonstrating the efficacy of IRES-targeted therapy in the tumor [40]. Although whether IRES is activated in the face of eIF4E-dependent (cap-dependent) translation inhibition by ATRA is unknown, IRES-dependent BCL-2 translation protected leukemic cells from programmed cell death, and we found that AML cells might protect themselves from ATRA-induced cell apoptosis by increasing the translation of BCL-2 in compensation. Synergistic therapy combing ATRA and IRES inhibitors is a promising strategy for AML patients and needs to be further elucidated. G4s, a stable secondary structure in 5′UTR, is also involved in RNA translation, DNA damage, and genomic instability. The optimal translation activity of G4s-mRNA translation was proved to be dependent on the helicase activity of eIF4A, another eIF4F complex component that was reduced by ATRA stress in our current study [41]. Opportune translation of mRNAs with G4s in 5′UTR (G4s-mRNAs) is required for hematopoiesis. Researchers have reported that blockage in G4s-mRNA translation, caused by failure to unfold due to helicase DHX36 depletion, resulted in hemolytic anemia and proerythroid defects [42]. Anti-apoptotic protein Aven has been identified to regulate the translation of mixed lineage leukemia (MLL) oncogene MLL1 and MLL4 by binding the G4s structure through its RGG/RG motif, and shRNA targeting Aven-decreased T-ALL cell proliferation [43]. Whether G4s is implicated in mRNA translation activity controlled by 5′UTR during ATRA stress remains to be investigated, but the development of therapy including an agent that can directly or indirectly inhibit helicase such as eIF4A is a benefit for leukemia patients, as the antiproliferative activity of synthetic organic heterocyclic small molecule ligands targeting G4s in HL60 and K562 has also been validated in vitro, and natural compound Silvestrol targeting eIF4A significantly suppresses tumor growth [44,45].

Many issues need to be addressed: (a) One gene can be translated into multiple proteins (or isoforms) to perform specific functions. For example, cell differentiation critical transcript factor C/EBPα can be translated into a truncated isoform P30 due to aberrant eIF4E expression, thereby losing its activity in inducing cell differentiation but gaining the function to increase cell proliferation [46]. Whether ATRA changes the selection of gene expression isoforms to create a translation landscape that benefits cell differentiation is unknown. (b) Notwithstanding that combining two or more treatments is beneficial for AML patients, the consequent toxicity should also be considered. In particular, the sequential strategy is target selectivity and time dependence, some technical methods such as quantitative pharmacology with the mathematical model, pharmacogenomic testing, or therapeutical drug monitoring (TDM) might be required before therapy. (c) We observed that a large amount of long noncoding RNAs might have the potential to be translated into peptides as they were detected in the RPFs, and further research is required to clarify the role of these peptides in AML therapy.

## 5. Conclusions

Collectively, we found that ATRA translationally reprogrammed AML cell development, and the translation initiator eIF4E was the critical regulator for AML therapy. Further investigation of ATRA-induced translatome revealed the abnormal activation of PI3K/AKT during ATRA treatment, and we then advised a sequential strategy containing ATRA plus a PI3K/AKT inhibitor for AML therapy.

## Data Availability

Not applicable.

## Supplementary Materials

The following supporting information can be downloaded at: https://www.mdpi.com/article/10.3390/pharmaceutics14112329/s1, Figure S1: ATRA and ATPR induced cell differentiation in Molm13 cells; Figure S2: ATRA and ATPR inhibited global translation and protein synthesis; Figure S3: eIF4E was a critical regulator in ATRA or ATPR-induced AML cell growth arrest and differentiation; Figure S4: ATRA translationally reprogrammed AML cell development; Figure S5: PI3K/AKT activation was required for ATRA and ATPR to induce AML cell differentiation; Figure S6: Sequential strategy combing PI3K/AKT inhibitor following ATRA or ATPR eliminated AML cells.

## References

[1] Vetrie D., Helgason G.V., Copland M. (2020). The leukaemia stem cell: Similarities, differences and clinical prospects in CML and AML. Nat. Rev. Cancer.

[2] Ding L., Ley T.J., Larson D.E., Miller C.A., Koboldt D.C., Welch J.S., Ritchey J.K., Young M.A., Lamprecht T., McLellan M.D. (2012). Clonal evolution in relapsed acute myeloid leukaemia revealed by whole-genome sequencing. Nature.

[3] de Rezende M.M., Ferreira A.T., Paredes-Gamero E.J. (2020). Leukemia stem cell immunophenotyping tool for diagnostic, prognosis, and therapeutics. J. Cell. Physiol..

[4] Liang C., Qiao G., Liu Y., Tian L., Hui N., Li J., Ma Y., Li H., Zhao Q., Cao W. (2021). Overview of all-trans-retinoic acid (ATRA) and its analogues: Structures, activities, and mechanisms in acute promyelocytic leukaemia. Eur. J. Med. Chem..

[5] McKenzie M.D., Ghisi M., Oxley E.P., Ngo S., Cimmino L., Esnault C., Liu R., Salmon J.M., Bell C.C., Ahmed N. (2019). Interconversion between Tumorigenic and Differentiated States in Acute Myeloid Leukemia. Cell Stem Cell.

[6] Wass M., Gollner S., Besenbeck B., Schlenk R.F., Mundmann P., Gothert J.R., Noppeney R., Schliemann C., Mikesch J.H., Lenz G. (2021). A proof of concept phase I/II pilot trial of LSD1 inhibition by tranylcypromine combined with ATRA in refractory/relapsed AML patients not eligible for intensive therapy. Leukemia.

[7] Perez-Ortin J.E., Tordera V., Chavez S. (2019). Homeostasis in the Central Dogma of molecular biology: The importance of mRNA instability. RNA Biol..

[8] Liu Y., Beyer A., Aebersold R. (2016). On the Dependency of Cellular Protein Levels on mRNA Abundance. Cell.

[9] Hershey J.W.B., Sonenberg N., Mathews M.B. (2019). Principles of Translational Control. Cold Spring Harb. Perspect. Biol..

[10] Wang K., Wang C., Zhu C.J., Li G., Li Y., Feng Y.B., Ruan J.J., Zhu F., Meng Y., Zhou R.P. (2018). 4-Amino-2-Trifluoromethyl-Phenyl Retinate induced leukemia cell differentiation by decreasing eIF6. Biochem. Biophys. Res. Commun..

[11] Li G., Wang K., Li Y., Ruan J., Wang C., Qian Y., Zu S., Dai B., Meng Y., Zhou R. (2019). Role of eIF3a in 4-amino-2-trifluoromethyl-phenyl retinate-induced cell differentiation in human chronic myeloid leukemia K562 cells. Gene.

[12] Borden K.L.B., Volpon L. (2020). The diversity, plasticity, and adaptability of cap-dependent translation initiation and the associated machinery. RNA Biol..

[13] Jia Y., Polunovsky V., Bitterman P.B., Wagner C.R. (2012). Cap-dependent translation initiation factor eIF4E: An emerging anticancer drug target. Med. Res. Rev..

[14] Barbieri I., Tzelepis K., Pandolfini L., Shi J., Millan-Zambrano G., Robson S.C., Aspris D., Migliori V., Bannister A.J., Han N. (2017). Promoter-bound METTL3 maintains myeloid leukaemia by m(6)A-dependent translation control. Nature.

[15] Szavits-Nossan J., Ciandrini L. (2020). Inferring efficiency of translation initiation and elongation from ribosome profiling. Nucleic Acids Res..

[16] Mali R.S., Zhang Q., DeFilippis R.A., Cavazos A., Kuruvilla V.M., Raman J., Mody V., Choo E.F., Dail M., Shah N.P. (2021). Venetoclax combines synergistically with FLT3 inhibition to effectively target leukemic cells in FLT3-ITD+ acute myeloid leukemia models. Haematologica.

[17] Merrick W.C., Pavitt G.D. (2018). Protein Synthesis Initiation in Eukaryotic Cells. Cold Spring Harb. Perspect. Biol..

[18] Davis M.R., Delaleau M., Borden K.L.B. (2019). Nuclear eIF4E Stimulates 3′-End Cleavage of Target RNAs. Cell Rep..

[19] Culjkovic-Kraljacic B., Fernando T.M., Marullo R., Calvo-Vidal N., Verma A., Yang S., Tabbo F., Gaudiano M., Zahreddine H., Goldstein R.L. (2016). Combinatorial targeting of nuclear export and translation of RNA inhibits aggressive B-cell lymphomas. Blood.

[20] Moerke N.J., Aktas H., Chen H., Cantel S., Reibarkh M.Y., Fahmy A., Gross J.D., Degterev A., Yuan J., Chorev M. (2007). Small-molecule inhibition of the interaction between the translation initiation factors eIF4E and eIF4G. Cell.

[21] de The H. (2018). Differentiation therapy revisited. Nat. Rev. Cancer.

[22] Geoffroy M.C., Esnault C., de The H. (2021). Retinoids in hematology: A timely revival?. Blood.

[23] Lucena-Araujo A.R., Kim H.T., Jacomo R.H., Melo R.A., Bittencourt R., Pasquini R., Pagnano K., Fagundes E.M., Mde L.C., Chiattone C.S. (2014). Internal tandem duplication of the FLT3 gene confers poor overall survival in patients with acute promyelocytic leukemia treated with all-trans retinoic acid and anthracycline-based chemotherapy: An International Consortium on Acute Promyelocytic Leukemia study. Ann. Hematol..

[24] Liu L., Yang L., Liu X., Liu M., Liu J., Feng X., Nie Z., Luo J. (2022). SEMA4D/PlexinB1 promotes AML progression via activation of PI3K/Akt signaling. J. Transl. Med..

[25] Dumas P.Y., Naudin C., Martin-Lanneree S., Izac B., Casetti L., Mansier O., Rousseau B., Artus A., Dufossee M., Giese A. (2019). Hematopoietic niche drives FLT3-ITD acute myeloid leukemia resistance to quizartinib via STAT5-and hypoxia-dependent upregulation of AXL. Haematologica.

[26] Feng Y., Hua X., Niu R., Du Y., Shi C., Zhou R., Chen F.H. (2019). ROS play an important role in ATPR inducing differentiation and inhibiting proliferation of leukemia cells by regulating the PTEN/PI3K/AKT signaling pathway. Biol. Res..

[27] Shim G., Kim M.G., Kim D., Park J.Y., Oh Y.K. (2017). Nanoformulation-based sequential combination cancer therapy. Adv. Drug Deliv. Rev..

[28] Pandey A., Kulkarni A., Roy B., Goldman A., Sarangi S., Sengupta P., Phipps C., Kopparam J., Oh M., Basu S. (2014). Sequential application of a cytotoxic nanoparticle and a PI3K inhibitor enhances antitumor efficacy. Cancer Res..

[29] Yoon H.Y., Son S., Lee S.J., You D.G., Yhee J.Y., Park J.H., Swierczewska M., Lee S., Kwon I.C., Kim S.H. (2014). Glycol chitosan nanoparticles as specialized cancer therapeutic vehicles: Sequential delivery of doxorubicin and Bcl-2 siRNA. Sci. Rep..

[30] Suchorska W.M., Augustyniak E., Lukjanow M. (2016). Genetic stability of pluripotent stem cells during anti-cancer therapies. Exp. Ther. Med..

[31] Wen P.Y., Touat M., Alexander B.M., Mellinghoff I.K., Ramkissoon S., McCluskey C.S., Pelton K., Haidar S., Basu S.S., Gaffey S.C. (2019). Buparlisib in Patients with Recurrent Glioblastoma Harboring Phosphatidylinositol 3-Kinase Pathway Activation: An Open-Label, Multicenter, Multi-Arm, Phase II Trial. J. Clin. Oncol..

[32] Baselga J., Im S.A., Iwata H., Cortes J., de Laurentiis M., Jiang Z., Arteaga C.L., Jonat W., Clemons M., Ito Y. (2017). Buparlisib plus fulvestrant versus placebo plus fulvestrant in postmenopausal, hormone receptor-positive, HER2-negative, advanced breast cancer (BELLE-2): A randomised, double-blind, placebo-controlled, phase 3 trial. Lancet Oncol..

[33] Rosenthal M., Clement P.M., Campone M., Gil-Gil M.J., DeGroot J., Chinot O., Idbaih A., Gan H., Raizer J., Wen P.Y. (2020). Buparlisib plus carboplatin or lomustine in patients with recurrent glioblastoma: A phase Ib/II, open-label, multicentre, randomised study. ESMO Open.

[34] Soulieres D., Faivre S., Mesia R., Remenar E., Li S.H., Karpenko A., Dechaphunkul A., Ochsenreither S., Kiss L.A., Lin J.C. (2017). Buparlisib and paclitaxel in patients with platinum-pretreated recurrent or metastatic squamous cell carcinoma of the head and neck (BERIL-1): A randomised, double-blind, placebo-controlled phase 2 trial. Lancet Oncol..

[35] Leppek K., Das R., Barna M. (2018). Functional 5′ UTR mRNA structures in eukaryotic translation regulation and how to find them, Nature reviews. Mol. Cell Biol..

[36] King H.A., Cobbold L.C., Willis A.E. (2010). The role of IRES trans-acting factors in regulating translation initiation. Biochem. Soc. Trans..

[37] Muranen T., Selfors L.M., Worster D.T., Iwanicki M.P., Song L., Morales F.C., Gao S., Mills G.B., Brugge J.S. (2012). Inhibition of PI3K/mTOR leads to adaptive resistance in matrix-attached cancer cells. Cancer Cell.

[38] Emadi A., Kapadia B., Bollino D., Bhandary B., Baer M.R., Niyongere S., Strovel E.T., Kaizer H., Chang E., Choi E.Y. (2021). Venetoclax and pegcrisantaspase for complex karyotype acute myeloid leukemia. Leukemia.

[39] Kampen K.R., Sulima S.O., Verbelen B., Girardi T., Vereecke S., Rinaldi G., Verbeeck J., de Beeck J.O., Uyttebroeck A., Meijerink J.P.P. (2019). The ribosomal RPL10 R98S mutation drives IRES-dependent BCL-2 translation in T-ALL. Leukemia.

[40] Vaklavas C., Grizzle W.E., Choi H., Meng Z., Zinn K.R., Shrestha K., Blume S.W. (2016). IRES inhibition induces terminal differentiation and synchronized death in triple-negative breast cancer and glioblastoma cells. Tumour Biol..

[41] Wolfe A.L., Singh K., Zhong Y., Drewe P., Rajasekhar V.K., Sanghvi V.R., Mavrakis K.J., Jiang M., Roderick J.E., van der Meulen J. (2014). RNA G-quadruplexes cause eIF4A-dependent oncogene translation in cancer. Nature.

[42] Lai J.C., Ponti S., Pan D., Kohler H., Skoda R.C., Matthias P., Nagamine Y. (2012). The DEAH-box helicase RHAU is an essential gene and critical for mouse hematopoiesis. Blood.

[43] Thandapani P., Song J., Gandin V., Cai Y., Rouleau S.G., Garant J.M., Boisvert F.M., Yu Z., Perreault J.P., Topisirovic I. (2015). Aven recognition of RNA G-quadruplexes regulates translation of the mixed lineage leukemia protooncogenes. eLife.

[44] Zhang W., Gong P., Tian Q., Han S., Wang J., He P., Guo Y., Wang G., Chen Q., Huang J. (2022). The eIF4A Inhibitor Silvestrol Blocks the Growth of Human Glioblastoma Cells by Inhibiting AKT/mTOR and ERK1/2 Signaling Pathway. J. Oncol..

[45] Das R.N., Chevret E., Desplat V., Rubio S., Mergny J.L., Guillon J. (2017). Design, Synthesis and Biological Evaluation of New Substituted Diquinolinyl-Pyridine Ligands as Anticancer Agents by Targeting G-Quadruplex. Molecules.

[46] Calkhoven C.F., Muller C., Leutz A. (2000). Translational control of C/EBPalpha and C/EBPbeta isoform expression. Genes Dev..

