# Supplementary material for: The Translational Landscape Revealed the Sequential Treatment Containing ATRA plus PI3K/AKT Inhibitors as an Efficient Strategy for AML Therapy
Source: Pharmaceutics. 2022 Oct 28;14(11):2329. doi: 10.3390/pharmaceutics14112329 (PMC9696193; doi:10.3390/pharmaceutics14112329)
Supplement: Supplementary file 1 [file pharmaceutics-14-02329-s001.zip › Supplemental Figure legends.pdf]

Supplemental Figure Legends

Figure S1. ATRA and ATPR induced cell differentiation in Molm13 cells.

Flow cytometry was used to analyzed the proportion of CD11b-positive Molm13 cells after ATRA (A) and ATPR (B) treatment at the indicated times. $^{*}P < 0.05$, $^{**}P < 0.01$ versus control; bar, 100 μm.

Figure S2. ATRA and ATPR inhibited global translation and protein synthesis.

A. Total protein content was extracted from Molm13 cells after ATRA or ATPR treatment and then stained by coomassie brilliant blue assay. B. Immunofluorescent staining of NB4 cells showing expression of eIF4E (Green) with DAPI (Blue). C. Western blot analysis showing protein level of eIF4G, eIF4A, eIF4EBP1 (4EBP1), and phosphorylation level of 4EBP1 at ser65 and Thr37/46 in NB4 cells treating with ATRA or ATPR. D. Western blot analysis showing the distribution of eIF4E in cytoplasmic and nuclear of NB4 cells with ATRA or ATPR exposure. β-actin served as a loading control. β-actin and histone 3 serve as loading controls; $^{*}P < 0.05$, $^{**}P < 0.01$ versus control; bar, 100 μm.

Figure S3. eIF4E was a critical regulator in ATRA or ATPR-induced AML cell growth arrest and differentiation.

A and B. Validation of eIF4E knockdown was assessed by qPCR (A) and western blot (B). C. CCK8 assay were used to detect cell viability. D and E. Cell proliferation was analyzed by the fluorescence intensity of Ki67 (D) and the protein level of PCNA, CDK2 and Cyclin D1 (E). F. The percentage of CD11b-postive cells was used as a measure of cell differentiation and

detected by flow cytometry. G. Cell morphology was illustrated by wright-giemsa staining with a inverted microscope. H and I. Validation of eIF4E overexpression was assessed by qPCR (H) and western blot (I). *$P < 0.05$, **$P < 0.01$ versus control; bar, 100 μm.

Figure S4. ATRA translationally reprogrammed AML cell development.

GO enrichment were performed to analyze molecular function (A), cellular components (B), and biological process (C).

Figure S5. PI3K/AKT activation was required for ATRA and ATPR to induce AML cell differentiation.

Molm13 cells were treated with ATRA (A) or ATPR (B) and PI3K/AKT inhibitor LY294002 simultaneously. The percentage of CD11b-postive cells was used as a measure of cell differentiation and detected by flow cytometry.

Figure S6. Sequential strategy combing PI3K/AKT inhibitor following ATRA or ATPR eliminated AML cells.

Molm13 cells were pre-treated with ATRA or ATPR and then released into LY294002 exposure. A. Cell apoptosis were detected by flow cytometry using an Annexin V-FITC/PI staining kit. B. TUNEL analysis was processed to further measure the degree of cell apoptosis, and flow cytometry was utilized to quantify the cell apoptosis. C and D. The self-renewal capacity of Molm13 cells was analyzed by cell clonality and illustrated by colony forming unit (CFU) assay. Image J was employed to quantify the CFU data (C, ATRA; D, ATPR). E. The

procedure of $CD34^+$ HSPCs isolation from cord blood. F. Schematic diagram of FLT3-ITD driven transformation of $CD34^+$ HSPCs.
