# Supplementary figures and images for: The Translational Landscape Revealed the Sequential Treatment Containing ATRA plus PI3K/AKT Inhibitors as an Efficient Strategy for AML Therapy
Source: Pharmaceutics. 2022 Oct 28;14(11):2329. doi: 10.3390/pharmaceutics14112329 (PMC9696193; doi:10.3390/pharmaceutics14112329)

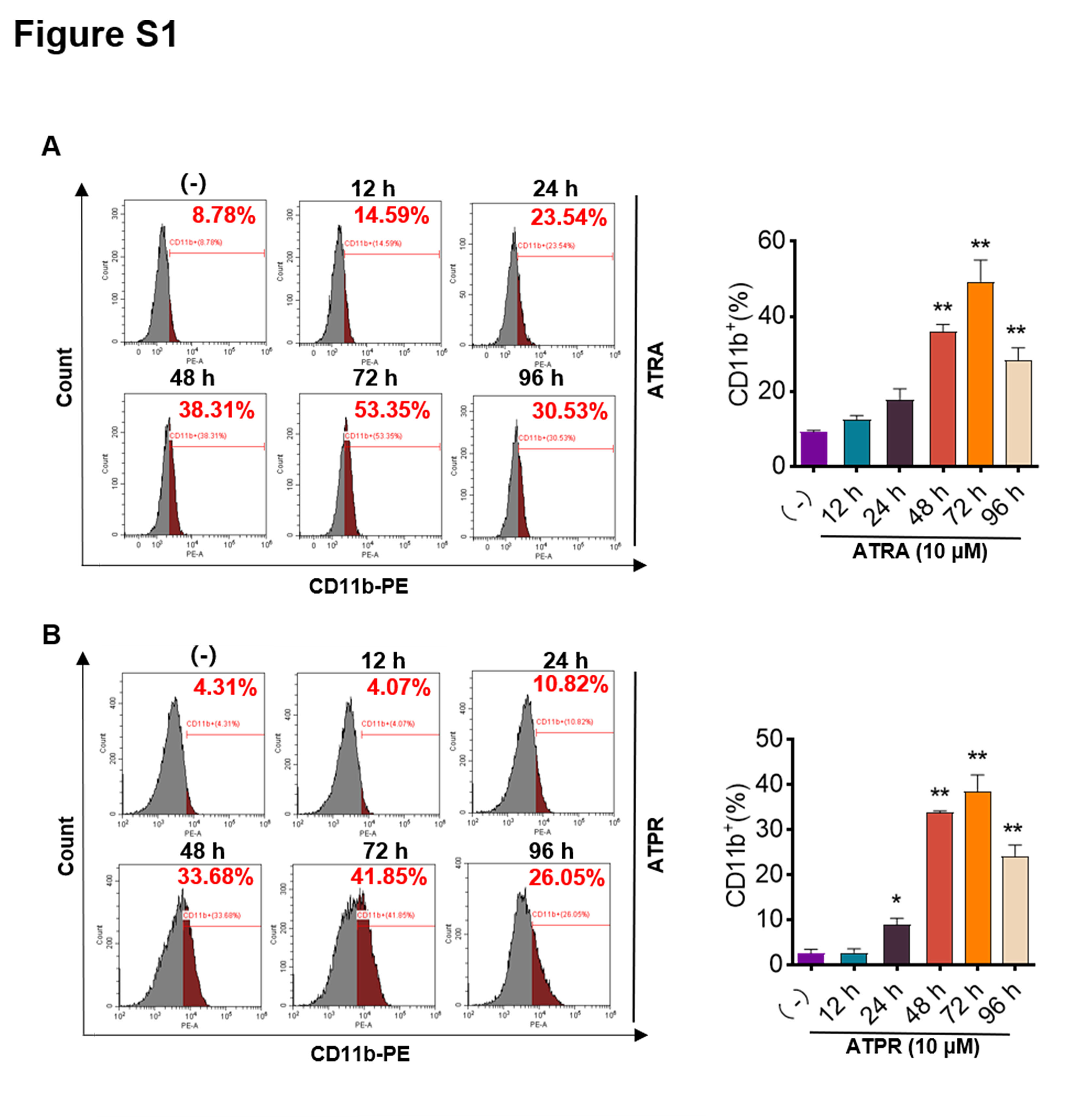

Supplement: Supplementary file 1 [file pharmaceutics-14-02329-s001.zip › Supplemental figures/Figure S1.jpg]

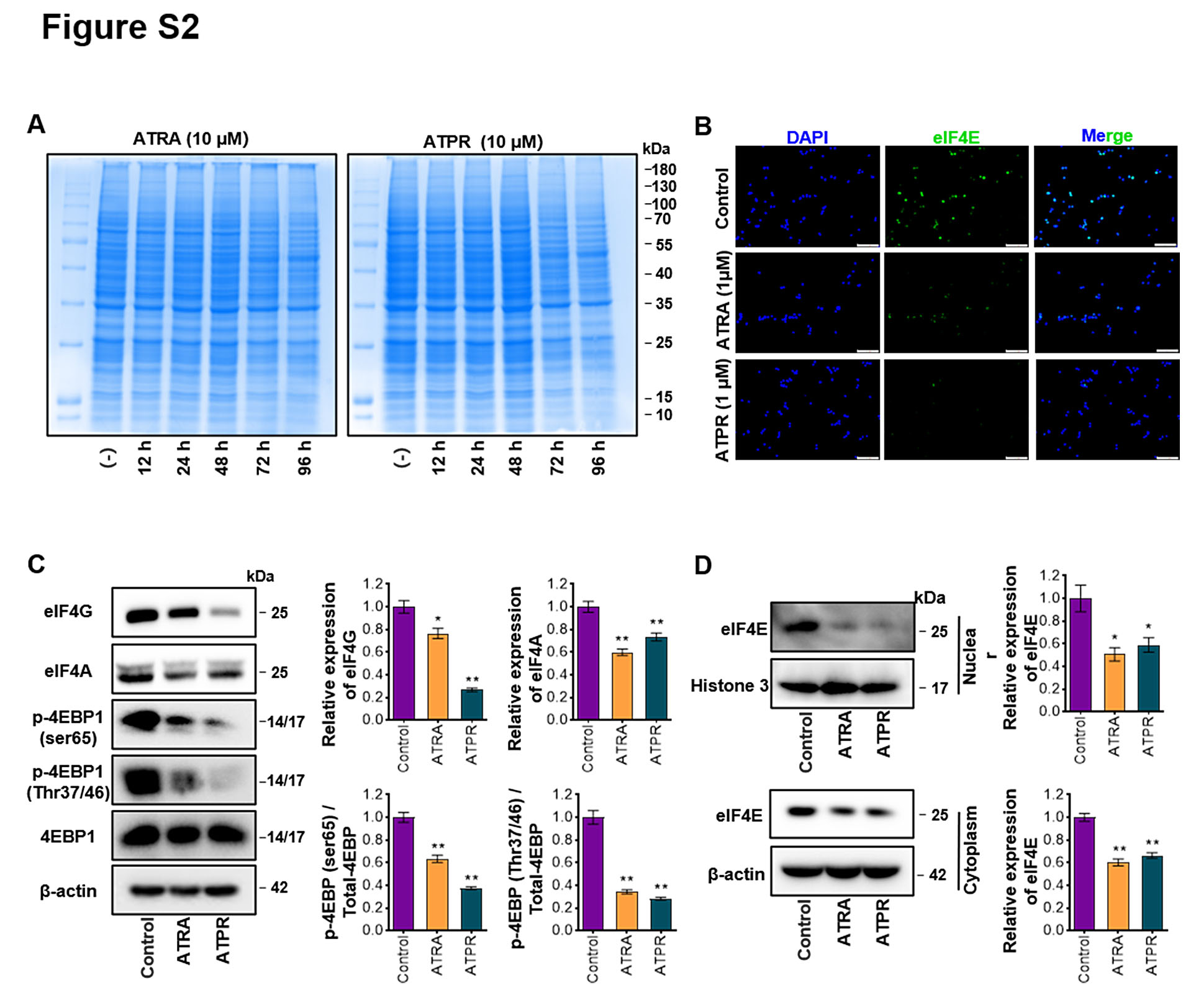

Supplement: Supplementary file 1 [file pharmaceutics-14-02329-s001.zip › Supplemental figures/Figure S2.jpg]

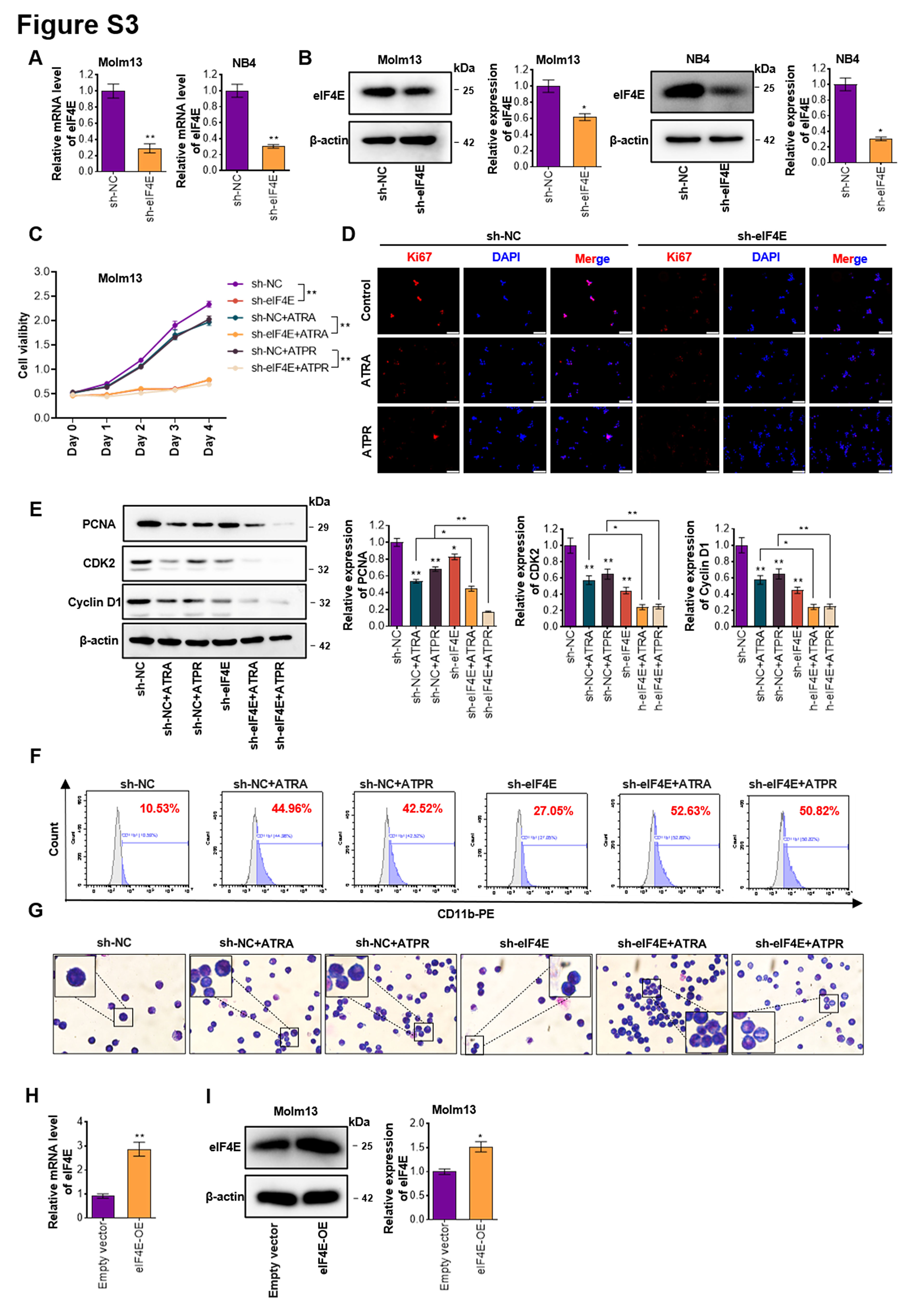

Supplement: Supplementary file 1 [file pharmaceutics-14-02329-s001.zip › Supplemental figures/Figure s3.jpg]

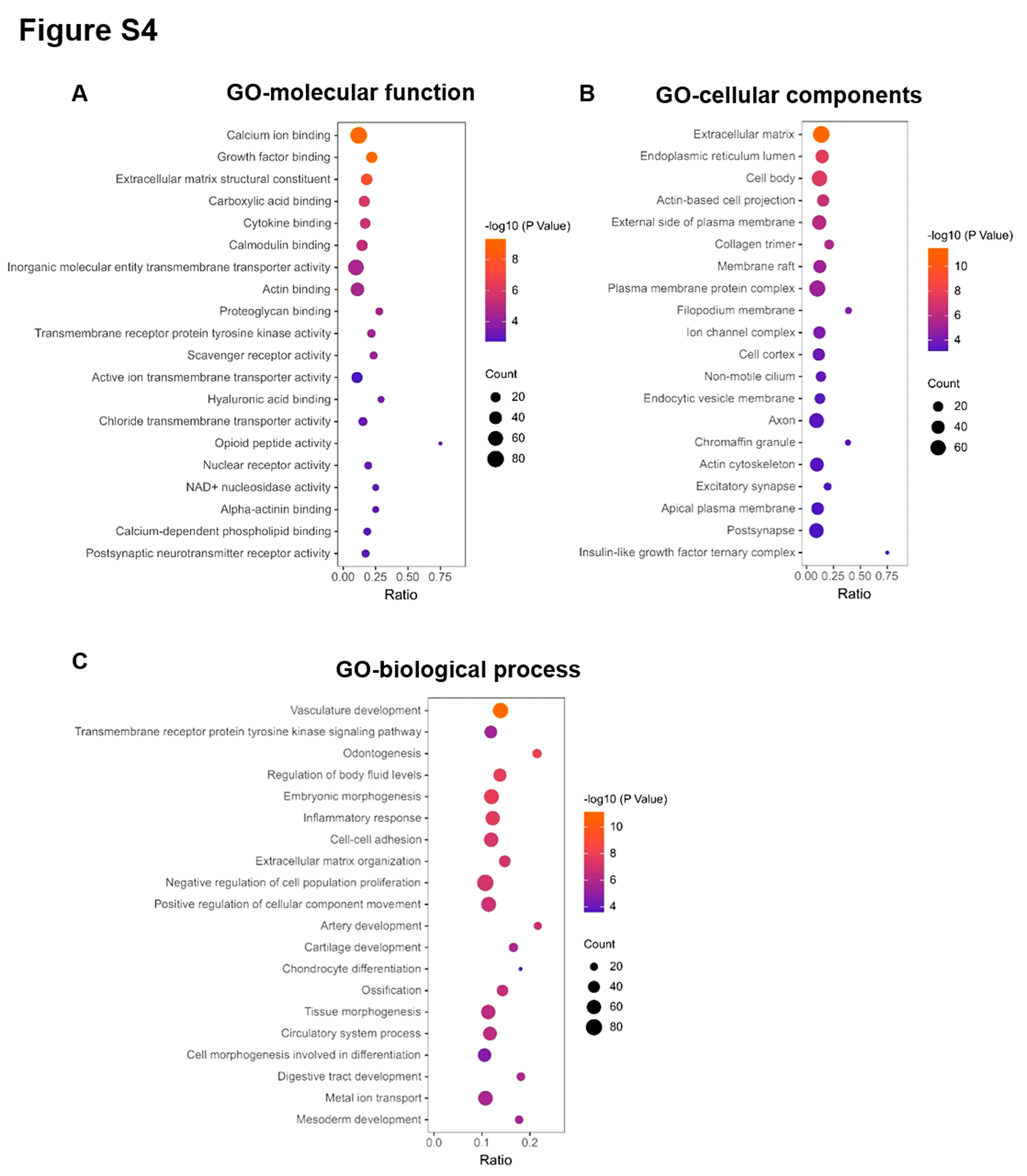

Supplement: Supplementary file 1 [file pharmaceutics-14-02329-s001.zip › Supplemental figures/Figure S4.jpg]

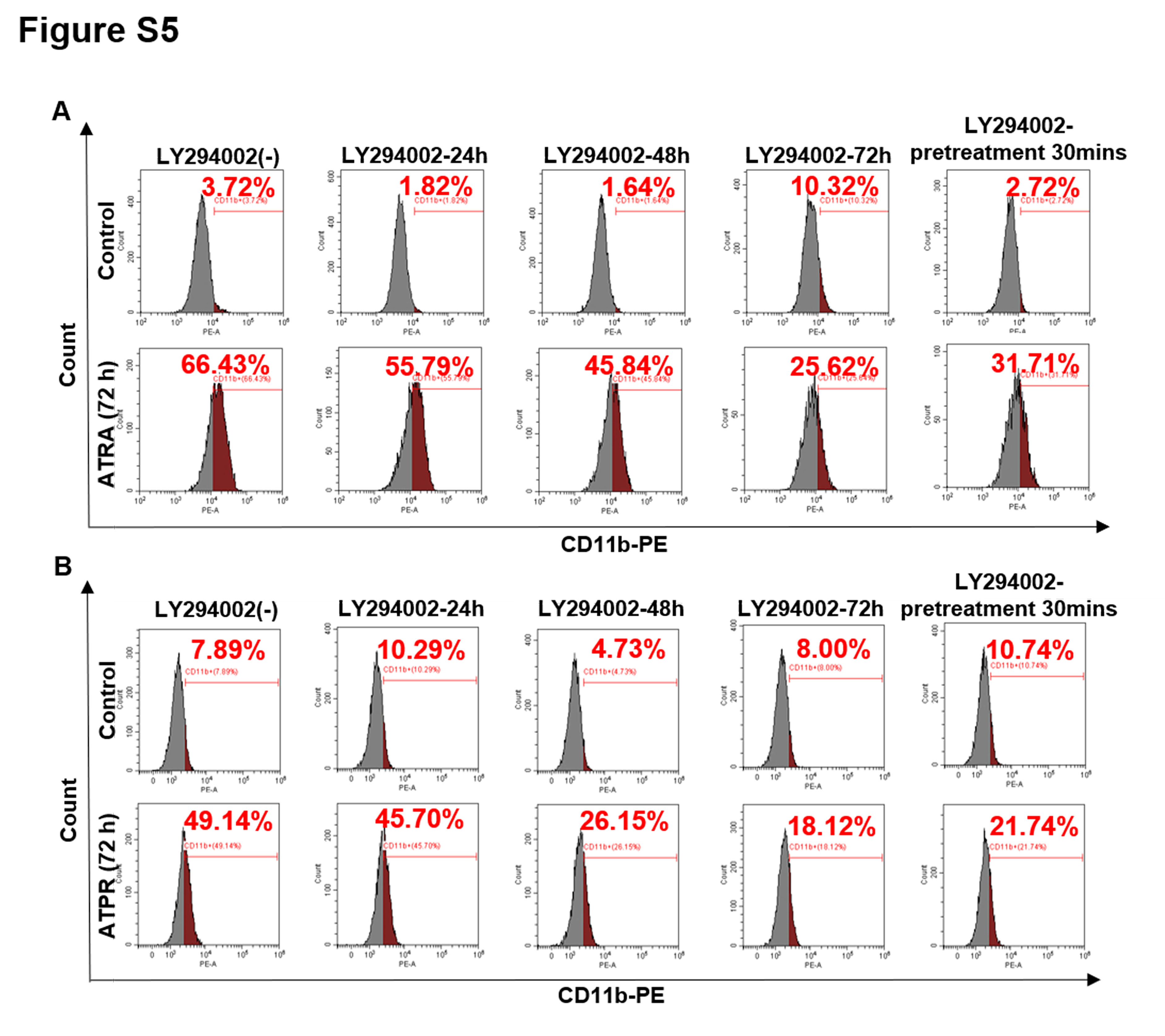

Supplement: Supplementary file 1 [file pharmaceutics-14-02329-s001.zip › Supplemental figures/Figure S5.jpg]

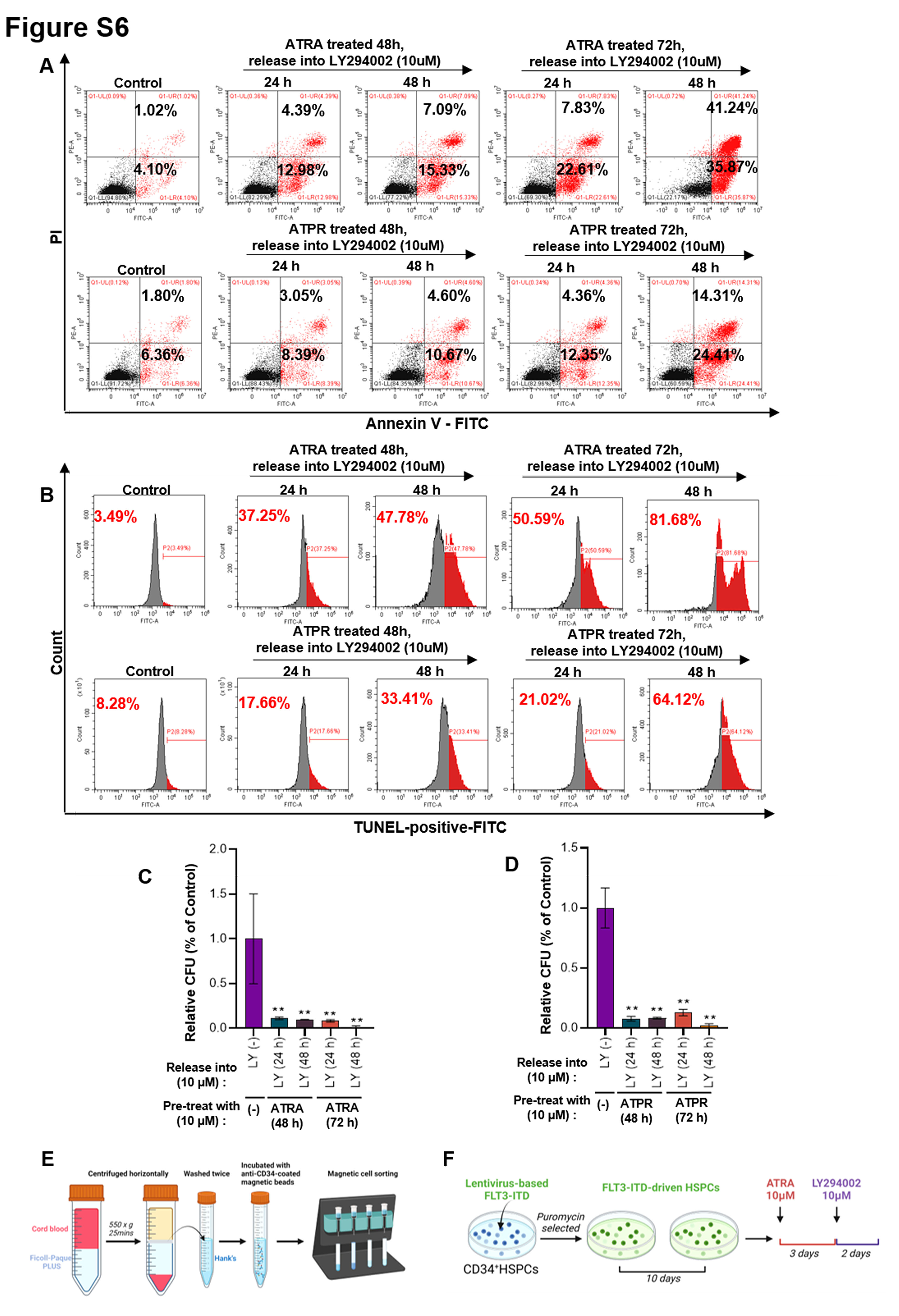

Supplement: Supplementary file 1 [file pharmaceutics-14-02329-s001.zip › Supplemental figures/Figure S6.jpg]
